# Soluble Urokinase Receptor and the Kidney Response in Diabetes Mellitus

**DOI:** 10.1155/2017/3232848

**Published:** 2017-05-17

**Authors:** Ranadheer R. Dande, Vasil Peev, Mehmet M. Altintas, Jochen Reiser

**Affiliations:** Rush University Medical Center, Chicago, IL, USA

## Abstract

Diabetic nephropathy (DN) is the leading cause of end-stage renal disease (ESRD) worldwide. DN typically manifests by glomerular hyperfiltration and microalbuminuria; then, the disease progresses to impaired glomerular filtration rate, which leads to ESRD. Treatment options for DN include the strict control of blood glucose levels and pressure (e.g., intraglomerular hypertension). However, the search for novel therapeutic strategies is ongoing. These include seeking specific molecules that contribute to the development and progression of DN to potentially interfere with these “molecular targets” as well as with the cellular targets within the kidney such as podocytes, which play a major role in the pathogenesis of DN. Recently, podocyte membrane protein urokinase receptor (uPAR) and its circulating form (suPAR) are found to be significantly induced in glomeruli and sera of DN patients, respectively, and elevated suPAR levels predicted diabetic kidney disease years before the occurrence of microalbuminuria. The intent of this review is to summarize the emerging evidence of uPAR and suPAR in the clinical manifestations of DN. The identification of specific pathways that govern DN will help us build a more comprehensive molecular model for the pathogenesis of the disease that can inform new opportunities for treatment.

Diabetes mellitus (DM) is a disorder of glucose metabolism that occurs due to either defect in insulin production by the pancreatic beta cells (type 1 DM) or resistance to insulin in the peripheral tissues (type 2 DM). With the increasing prevalence in obesity and metabolic syndrome, incidence of type 2 DM has been increasing worldwide, including the United States, where approximately 29.1 million people or 9.3% of the population are affected [1]. It is estimated that more than 400 million people will be affected with DM by 2030 [2]. Urinary albumin excretion ranging between 30 and 300 mg/24 h (microalbuminuria) is the earliest sign of diabetic kidney disease (DKD) [3]. Along with microalbuminuria, DN is also characterized by the increased levels of plasma creatinine and the decreased estimated glomerular filtration rate (eGFR) [4] since almost one third of type 2 diabetes patients have renal insufficiency without microalbuminuria [5]. This alone questions the assumption that microalbuminuria could be used as a marker rather than a predictor of DN [6]. Diabetic nephropathy (DN) is the major microvascular complication of diabetes and is one of the leading causes of end-stage renal disease (ESRD) affecting one third of all diabetic individuals in the United States [7]. Persistently high albumin excretion (≥300 mg/24 h), a condition known as macroalbuminuria, increases the chances of progressing to ESRD by 10 times compared to patients with normal urine albumin levels [8]. Many factors including diet, lifestyle, chronic blood glucose levels (HbA1C), blood pressure (BP), smoking, serum cholesterol, and genetic predisposition together play a crucial role in the progression of DN to ESRD.

Since the renin–angiotensin system (RAS) plays an important role in regulating systemic BP, blockade of its activation by either angiotensin-converting enzyme inhibitors (ACEIs) or angiotensin II type 1 receptor blockers (ARBs) are standard treatments for lowering the BP as well as slowing the progression of DN [9] and chronic renal failure [10]. Cumulative evidence has demonstrated that these first-line agents have represented a significant benefit in regard to partial renal protection in patients with diabetes and proteinuria [11–13]. However, antihypertensive therapy with RAS blockers contributes to the hyperkalemia (high potassium level in the blood) [14] especially when the patients treated with a combined therapy utilizing both an ACE inhibitor and an ARB together [15]. Other concerns of the RAS blockade include the potential long-term adverse effects and the need for dose optimization or individualization [16].

Recently, soluble urokinase receptor (suPAR) has been associated with podocytopathy, FSGS, and systemic levels of suPAR which are increased in patients with DM. Elevated suPAR levels predict incident chronic kidney disease also in patients with DM [17]. Furthermore, elevated suPAR in healthy people with predisposition for DM predicts microalbuminuria, an established early sign of DN by several years [18]. Podocytes have a major role in the pathogenesis of DN and its progression to ESRD [19, 20]. Podocytes are the important components of the glomerular filtration barrier that prevents the excretion of albumin into the urine [21]. These visceral epithelial cells of the glomerular tuft are a highly specialized structure containing a cell body, major processes that extend outward, and distal foot processes (FPs) that surround the glomerular capillaries [22]. Podocyte FPs are interconnected by a tiny multiprotein complex, slit diaphragm (SD), which regulates this active contractile structure ([23]). Podocytes sit on the glomerular basement membrane (GBM) [24], face the urinary space of Bowman's capsule, and form a unique filtration apparatus by interdigitating with the neighboring FPs. GBM separates podocytes from the innermost component of the glomerular filtration, endothelial cells, which are perforated by pores (“fenestrae” or “fenestrations”) to enhance the permeability of water and small solutes while still restricting the free passage of cellular components of blood to Bowman's (urinary) space [25]. Damage to the structural and functional components of podocytes results in the effacement of FPs (also referred to as “podocyte fusion” or “retraction”) and detachment of podocytes from the GBM causing the leak of serum proteins into the urine, a condition known as “proteinuria” [26, 27]. Proteinuria is a predictor of glomerular damage and hallmark of many renal disorders, including human and experimental DN. In various diabetic animal studies, it has been observed that there is podocyte hypertrophy (increase in the mean podocyte volume) [28, 29], cytoskeleton abnormalities [30, 31], and aberrant decrease in the density of podocytes [29, 32, 33]. Clinical studies were able to confirm the correlation between the podocyte loss and the disease progression in type 1 [34, 35] and type 2 diabetic patients [36–39] or in obesity-related glomerulopathy [40], which shares common pathophysiological factors relevant to glomerular damage with DN. Since podocytes have a limited capacity to divide [41], detached podocytes cannot be replaced by the adjacent podocytes; instead, surviving podocytes demonstrate an adaptation process called hypertrophy, where FPs of these residual podocytes enlarge to cover the surface of the glomerular capillary loops [22, 42]. Urinalysis of diabetic patients has shown that podocytes were seen in the urine of 53% and 80% of microalbuminuria and macroalbuminuria patients, respectively [43]. It should also be noted that podocytes remain viable after detachment and can be recovered in the urine pellets [44, 45]. Therefore, it comes as little surprise that podocytes are the key determinants of outcome for DKD and draw increased attention in diabetes research [46–51].

Owing to its complex architecture and dynamic movements, podocyte function is dependent on its abundantly rich actin cytoskeleton and ability to maintain diligently orchestrated interactions with the other members of the filtration barrier, that is, GBM and endothelial cells. The glomerular filtration barrier is permanently exposed to hydrostatic pressure gradient across the capillaries, and therefore, the integrity of the interaction between the podocytes and the GBM is essential for the filtration to occur. Podocytes, which are the most vulnerable components of the glomerular filtration, adhere to the underlying GBM via cell-matrix adhesion receptors, including *α*-dystroglycan; syndecan-4; type XVII collagen; integrins *α*3*β*1, *α*2*β*1, and *α*v*β*3; and a variety of other linker, scaffolding, and signaling proteins [52]. Among those, integrin family of cell adhesion receptors are of significant interest since they facilitate the interaction of podocytes with the extracellular matrix (ECM) at focal adhesions (FAs) and, as a separate regulatory function, transduce signals to the inside of the podocytes (outside-in signaling) to activate intracellular signaling events [53, 54]. The latter happens upon interaction of integrins with their respective ligands such as fibronectin, vitronectin, collagen, and laminin. This ligand binding process is not strictly receptor-specific, that is, each of these cell adhesion proteins can bind to more than one type of integrin. This receptor versatility generates a venue for a wide variety of intracellular signals required for development, growth, proliferation, differentiation, motility, cellular metabolism, and survival. In order to propagate intracellular signaling cascades, integrins need to connect actin cytoskeleton by recruiting a small repertoire of linker proteins such as paxillin, talin, vinculin, and *α*-actinin [55, 56] as the short cytoplasmic tails of these multi-subunit proteins lack actin-binding capacity [57], the only exception being integrin *β*4, which has ~1000 amino acids in its cytoplasmic tail (compared to that of a typical integrin *β* subunit, which is less than 75 amino acids long) and connects to the keratin cytoskeleton specifically [58]. The interaction with the adaptor proteins is mainly regulated by *β* subunits, whereas *α* subunits usually mediate the binding to ECM [59]. Given the emphasis on signaling, it is anticipated that integrins undergo significant conformational changes upon intracellular signaling leading to “integrin activation” that alters its ligand binding activity (inside-out signaling) [60, 61]. This mechanism highlights the bidirectional control of integrins' signaling [57, 62].

In humans, 24 *αβ* transmembrane heterodimers have been identified, making the integrin superfamily one of the most structurally diverse molecules of cell adhesion [63, 64]. *α*3*β*1 is the most highly expressed integrin in the kidney and the major regulator of the cell-matrix adhesion in podocytes [65, 66]. Mice deficient of *α*3*β*1 integrin failed to have the normal network of FPs; instead, displayed flattened podocyte processes that were still attached to GBM leading to decreased capillary loop formation [67] suggesting that *α*3*β*1 is needed for the proper rearrangement of podocyte cytoskeleton. A recent study showed that integrin *α*3*β*1 expression was upregulated in podocytes of patients with early DN [68] as opposed to the previous findings reporting a decrease in *α*3*β*1 expression in podocytes cultured under high-glucose conditions [69, 70] as well as in podocytes of animal models of DN [71–73] and DN patients [72]. On the other hand, expression of another member of integrin family, *α*v*β*3, increases in the setting of DN [74–76]. Taken together, these papers present a panoramic picture suggestive of the contribution of altered interaction of podocytes with GBM proteins to diabetic renal pathology. Major gaps in our understanding remain, particularly the molecular mechanisms by which hyperglycemia suppresses or upregulates the expression of these transmembrane receptors.

Our group recently reported that the *β*3 subunit of integrin was essential for the action of podocyte membrane protein urokinase plasminogen activator receptor (uPAR), which eventually led to effacement of the FPs and proteinuria [77]. uPAR lacks transmembrane and intracellular domains, and it is associated with the external surface of the plasma membrane by a glycosylphosphatidylinositol (GPI) anchor [78]. uPAR regulates the plasminogen activation system by binding urokinase (uPA) and its zymogen form, pro-UPA [79]. The ECM protein vitronectin is another immediate binding partner of uPAR and interacts with integrin coreceptors to activate integrin signaling and promote cell–ECM interactions [80, 81]. The resulting changes in expression or regulation of ECM receptors further instigate downstream signaling events that facilitate cell migration through activation of Rho family small GTPase Rac1 [77, 82, 83]. Interestingly, uPAR is not expressed in normal kidneys [84], and both uPA and uPAR expression is significantly upregulated in kidney cortex [85] and in all types of glomerular cells including podocytes [86, 87] in the animal models of DN.

Cell surface uPAR can be shed by several proteases, leaving it devoid of the GPI anchor, to generate a soluble form of uPAR (suPAR) [88, 89]. suPAR is a stable three-domain (D1, D2, and D3) protein that retains most of uPAR activities; both uPAR and suPAR are involved in the cell attachment, motility, and migration through their interaction with the integrins [90, 91]. Further cleavage through the linker connecting D1 and D2 domains generates a soluble D1 fragment and the residual D2-D3 fragment, which may remain membrane-bound or detach from the membrane [92, 93]. suPAR circulates in blood and other body fluids and has been identified in various pathological conditions: elevated plasma suPAR levels are predictive of cancer [94–96], cardiovascular disease (CVD) [94, 97–99], chronic kidney disease (CKD) [17], and type 1 [100] and type 2 diabetes [94, 101, 102]. In patients with type 2 DM, suPAR levels are increased with decreasing GFR, increasing proteinuria and can be a potential biomarker for staging of DN in type 2 DM patients [103]. Serum suPAR levels are also elevated in type 1 diabetic patients with DKD [104]. RAS blockade is an established treatment shown to decrease the progression of DN. Diabetic rats treated with ACEI resulted in inhibition of expression of uPAR in the kidneys [86]. In a randomized control study, RAS blockade in patients with DN showed decreased urinary suPAR levels compared to the group treated with placebo, but no difference is found in the plasma suPAR levels between the two groups [105]. This indicates that RAS blockade might decrease the renal suPAR production in vivo and can be the reason for decreased urinary suPAR levels in the treatment group compared to the placebo. With suPAR affecting the structure and functional aspects of podocytes resulting in FP effacement and proteinuria as well as decrease in the urinary level of suPAR by the treatment of renoprotective drugs like RAS blockers, there is a possibility that suPAR might have a major role in the pathogenesis and progression of DN. It remains to be determined what is the mechanism by which suPAR exerts its pathologic effects.

Our studies have demonstrated that, in LPS-treated mice, suPAR activates integrin *α*v*β*3 present on podocyte cell membrane in a similar manner to uPAR [77] and results in the podocyte FP effacement and podocyte migration leading to glomerular focal segmental glomerulosclerosis (FSGS) and proteinuria [106]. We recently reported that suPAR also activates integrin *α*v*β*3 when added to cultured human podocytes either in the form of suPAR-rich FSGS patient sera [106] or as a recombinant protein [104]. Of note, the pathological suPAR is originating from the bone marrow (BM) Gr-1^lo^ immature myeloid cells [107]. The suPAR-mediated podocyte injury can be prevented if suPAR activity is blocked by an uPAR-specific monoclonal antibody or by a small molecule that blocks *β*3 integrin activity, cycloRGDfV. It has been reported that efforts to inhibit uPA-uPAR protein interaction with a small molecule promotes conformational states of uPAR and weakens or even inhibits its interaction with vitronectin, a ligand of *β*3 integrin [108]. The functional relationship between *α*v*β*3 and uPAR/suPAR can also be interfered by acid sphingomyelinase-like phosphodiesterase 3b (SMPDL-3b) in the setting of DKD, where *α*v*β*3 integrin is not activated in the presence of high suPAR and increased SMPDL-3b, but the differential expression of these proteins rather causes podocyte motility and apoptosis [104]. Recently, blocking *α*v*β*3 integrin ligand occupancy by a monoclonal antibody that binds to *β*3 subunit of *α*v*β*3 integrin has been offered as a potential solution to prevent and reverse proteinuria associated with hyperglycemia in back-to-back studies [109, 110]. In these studies, it has been shown that targeting *α*v*β*3 with the monoclonal antibody alleviated several histological changes associated with DN in hyperglycemic pigs [109] and resulted in the reversal of proteinuria and inhibition of the synthesis of DN-related proteins in diabetic rat kidneys [110]. In the earlier related in vitro studies, the same group found out that the inhibition of ligand occupancy of *α*v*β*3 inhibited pathophysiologic (i.e., proliferative or migratory) responses of retinal endothelial [111], vascular endothelial, and smooth muscle cells [112] through a series of signaling pathways stimulated by insulin-like growth factor 1 (IGF-1) when the cells are exposed to the high glucose. Knowing that *α*v*β*3 integrin receptors are expressed in glomerular endothelium [75], similar events might occur in the glomeruli leading to endothelial permeability and proteinuria, which can be reversed by blocking *α*v*β*3 integrin.

In a recent prospective long-term cohort study of patients at risk for type 2 diabetes, it has been observed that higher baseline suPAR is independently associated with an increased risk of new-onset microalbuminuria and prediabetes [18]. The association of suPAR with incident microalbuminuria was reported to be independent from baseline eGFR. Importantly, the onset of microalbuminuria preceded the decline in GFR in this cohort. Moreover, suPAR predicted microalbuminuria irrespective of the baseline blood pressure and glycemia (e.g., baseline HbA1c). By these results, it can be hypothesized that suPAR can be an upstream biomarker for type 2 diabetes and also for DN even before the microalbuminuria. In another cross sectional study of patients with manifest type 2 diabetes, higher suPAR level is associated with high urinary albumin indicating that suPAR might be involved in the pathogenesis and progression of DN [18].

Overall, the transmembrane partnership between uPAR/suPAR and *α*v*β*3 integrin (as summarized in Figure 1) is an attractive target for the treatment of DKD. There are numerous studies dedicated to identify novel therapies efficiently targeting this delicate interaction with the use of antibodies, peptides, and small molecules. These efforts improve our understanding of the mechanism behind DN and our ability to predict the incidence of diabetic kidney disease (Figure 2), which will eventually advance the use of these agents toward clinical practice.

## Figures and Tables

**Figure 1:**
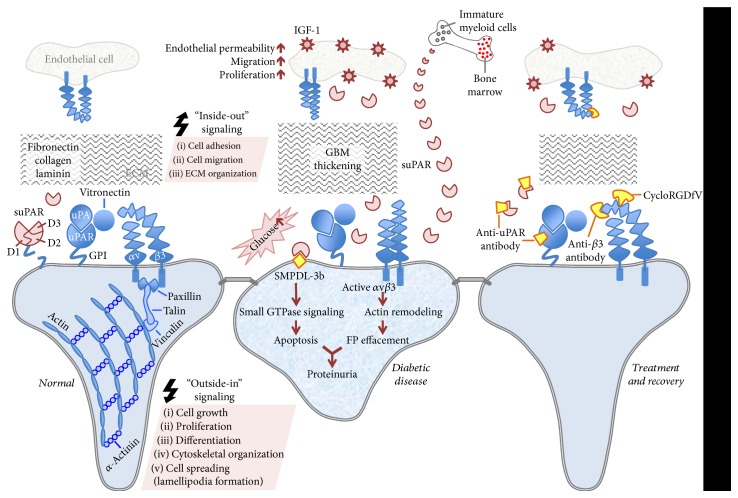
Schematic depiction of uPAR/suPAR-*α*v*β*3 integrin signaling at the glomerular filtration level in health and diabetic disease. In resting podocytes, uPAR interacts with uPA and anchored to the outer plasma membrane with GPI. This complex is connected to *α*v*β*3 integrin through vitronectin, a *β*3 integrin ligand. This leads to the initiation of “outside-in” signaling events, which requires the recruitment of linker proteins (paxillin, talin, and vinculin) by integrins for actin involvement. This signaling pathway is responsible for proper actin cytoskeleton assembly, lamellipodia formation, growth, proliferation, differentiation, and cell survival. ECM proteins such as fibronectin, collagen, and laminin are also involved in many cellular activities including ECM organization, cell adhesion and migration. Three homologous domains of uPAR are denoted by D1, D2, and D3, respectively (left panel). In the hyperglycemic state, *α*v*β*3 integrin activity increases causing altered adhesion, migration, and proliferation. These intracellular changes might initiate an “inside-out” signaling affecting integrin's binding affinity. Soluble uPAR also increases in circulation and probably contributes to the pathology of the diabetic kidney disease, which can be characterized as impaired cytoskeletal organization and podocyte FP effacement. The pathogenic suPAR is mainly generated by bone marrow-immature myeloid cells. Podocyte-specific expression of SMPDL-3b, which is elevated during the course of diabetic kidney disease, prevents *α*v*β*3 integrin activation by interacting with suPAR. This eventually increases RhoA activity and podocyte susceptibility to apoptosis. *α*v*β*3 integrin receptors are also expressed in glomerular endothelium and exposure of endothelial cells to hyperglycemia leads to pathologic outcomes in these cells such as endothelial permeability, migration, and proliferation in response to the ligand occupancy of *α*v*β*3 and concomitant stimulation of IGF-1 (middle panel). Targeting uPAR and suPAR with an uPAR-specific monoclonal antibody can attenuate the adverse effects of uPAR/suPAR-dependent integrin signaling. Using antibodies that bind preferentially to the activated and/or ligand-occupied forms of *β*3 integrin and *β*3 integrin small molecule inhibitor, cycloRGDfV, offer alternative ways to disentangle its interactions with uPAR/suPAR. Blocking the ligand occupancy of *α*v*β*3 inhibits the pathogenic mechanisms stimulated by IGF-1 (right panel).

**Figure 2:**
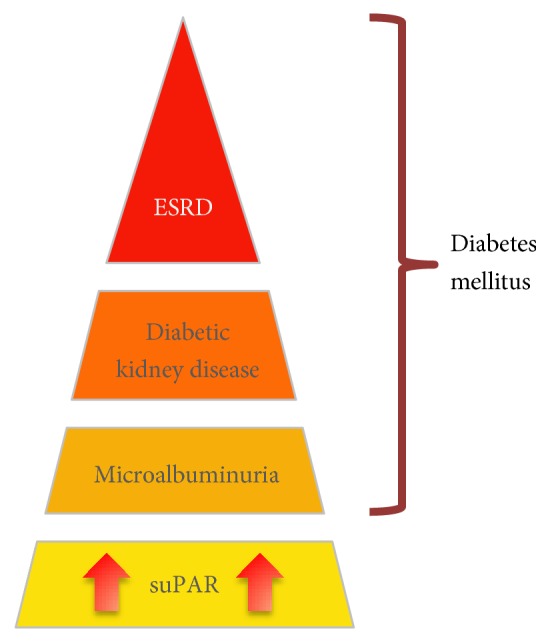
Schematic depiction of suPAR as a predictor for future diabetic kidney disease.
